# Bone Marrow Changes in Adolescent Girls With Anorexia Nervosa

**DOI:** 10.1359/jbmr.090805

**Published:** 2009-08-03

**Authors:** Kirsten Ecklund, Sridhar Vajapeyam, Henry A Feldman, Catherine D Buzney, Robert V Mulkern, Paul K Kleinman, Clifford J Rosen, Catherine M Gordon

**Affiliations:** 1 Department of Radiology, Children's Hospital Boston Boston, MA, USA; 2 Clinical Research Program, Children's Hospital Boston Boston, MA, USA; 3 Division of Adolescent/Young Adult Medicine, Children's Hospital Boston Boston, MA, USA; 4 Department of Medicine, Maine Medical Center Portland, ME, USA; 5 Division of Endocrinology, Children's Hospital Boston Boston, MA, USA

**Keywords:** magnetic resonance imaging, bone marrow, fat, relaxometry, anorexia nervosa

## Abstract

Early osteoporosis is common among adolescent girls with anorexia nervosa (AN) and may result from premature conversion of red (RM) to yellow bone marrow. We performed right knee magnetic resonance imaging (MRI) on a 1.0 T extremity scanner in 20 patients and 20 healthy controls, aged 16.2 ± 1.6 years (mean ± SD). Coronal T_1_-weighted (T_1_W) images and T_1_ maps were generated from T_1_ relaxometry images. Blinded radiologists visually assessed RM in the distal femoral and proximal tibial metaphyses in T_1_W images using a scale of signal intensity from 0 (homogeneous hyperintensity, no RM) to 4 (all dark, complete RM). Subjects with AN exhibited nearly twofold lower metaphyseal RM scores in both the femur (0.64 versus 1.22, *p* = .03) and tibia (0.54 versus 0.96, *p* = .08). In relaxometric measurements of four selected regions (femur and tibia amd epiphysis and metaphysis), subjects with AN showed higher mean epiphyseal but lower metaphyseal T_1_. The net AN-control difference between epiphysis and metaphysis was 70 ms in the femur (+31 versus −35 ms, *p* = .02) and of smaller magnitude in the tibia. In relaxometry data from the full width of the femur adjacent to the growth plate, AN subjects showed mean T_1_ consistently lower than in controls by 30 to 50 ms in virtually every part of the sampling region. These findings suggest that adolescents with AN exhibit premature conversion of hematopoietic to fat cells in the marrow of the peripheral skeleton potentially owing to adipocyte over osteoblast differentiation in the mesenchymal stem cell pool. © 2010 American Society for Bone and Mineral Research

## Introduction

Adolescent girls with anorexia nervosa (AN) are at risk for irreversible bone loss, early onset of osteoporosis, and an increased fracture risk compared with healthy adolescents.(1–6) The skeletal problems seen may be mechanistically linked to abnormalities in osteoblast and osteoclast progenitors within bone marrow.(7–10) Hormonal abnormalities in affected patients may mediate adipocyte over osteoblast differentiation in the mesenchymal stem cell pool, resulting in increased bone marrow fat and premature conversion to yellow marrow (YM). Examples include alterations in the secretion of estrogen, leptin, insulin-like growth factor 1 (IGF-1), growth hormone, and cortisol in response to weight loss that may mediate these changes.(11)

Previous reports examining bone marrow composition in adolescents with AN have yielded varying results.(12–19) Destructive morphologic changes and an alteration in bone marrow adipocytes have been observed, findings thought to be a direct result of severe weight loss in affected young women.(12) Two reports of magnetic resonance imaging (MRI) evaluations of bone marrow in these patients have shown a correlation between hematologic abnormalities, such as anemia and leukopenia, each a common finding in this patient group, and severe depletion of total-body fat mass.(15)(17) These studies suggest that bone marrow activity alternates between osteoblast and adipocyte formation, potentially linking increased marrow adiposity with bone loss and osteoporosis.(10)(20–23) MRI, computed tomography (CT), and bone marrow biopsy have been used previously to document hypoplasia of red hematopoietic marrow, reduced cellularity, and increased levels of YM in patients with AN in varying locations, including the lumbar vertebrae, pelvis, and femoral marrow spaces.(12–19) Similar lipid accumulation has been observed in aging individuals (ages 52 to 92 years) who suffer from osteoporosis.(24–30) Other studies examining marrow changes in young women with AN have shown an accumulation of extracellular hyaluronic acid in marrow spaces, a reduction of both YM and RM, and increased free water content.(13)(15) This hyperhydration and a reduced bone marrow fat fraction, observed via spectroscopy and relaxometry techniques, is suspected to be a physiologic response to reduced hematopoietic requirements as a result of decreased body mass.(13–14)(17)(31) As girls achieve peak bone mass during adolescence, disease-associated alterations in bone marrow composition and a subsequent inhibition of bone formation during this important developmental period may contribute to the increased fracture risk seen in these patients.

To our knowledge, bone marrow adiposity has not been investigated previously using MRI in adolescents with AN. This study aimed to investigate bone marrow composition in this young patient group and specifically to analyze the distribution of hemopoietic red marrow (RM) and fatty YM. We sought to understand the role of bone marrow in the impaired bone turnover and skeletal sequelae seen in adolescent girls with AN.

## Materials and Methods

### Subjects

Human subjects approval was obtained from the Committee on Clinical Investigation at Children's Hospital Boston. All participants provided written informed consent; for those under 18 years of age, a parent or guardian also provided consent. Female volunteers, ages 13 to 18 years (mean 16.3 years), were recruited prospectively from the adolescent outpatient clinic at Children's Hospital Boston. Each patient met diagnostic criteria for AN using the *Diagnostic and Statistical Manual of Mental Disorders*, Fourth Edition (DSM-IV). The patients had body mass indexes (BMIs) between 14.4 and 20.5 kg/cm^2^, had secondary amenorrhea of at least 3 months' duration, had no history of illness affecting bone metabolism, and had received no glucocorticoid therapy or other hormonal treatment within the past 3 months. Twenty healthy controls were obtained through the Children's Hospital Boston outpatient adolescent program or a Craigslist.com posting. These patients similarly had no history of illness affecting bone metabolism and had received no glucocorticoid therapy or hormonal treatment within the past 3 months. The controls had BMIs between 18.8 and 25.5 kg/m^2^. Adolescents with AN were matched with controls of the same race and pubertal stage who were within 2 years of the patient's chronologic age.

### Procedure

Weight and height data were obtained using a single calibrated scale (Tronix, Carol Steam, IL, USA) and stadiometer (Detecto, Webb City, MO, USA). BMI was calculated as weight in kilograms divided by the square of height in meters. An human chorionic gonadotropin (hCG) pregnancy test (SA Scientific, Ltd., San Antonio, TX, USA) was performed after consent had been obtained. A semistructured interview was conducted to obtain information about each patient's medical and bone fracture history, daily dietary intake, and level of physical activity. For patients with AN, severity of illness was assessed using self-reported information on duration of disease, if and how many times she had been hospitalized for AN (medical and psychiatric), and the date and numbers of her lowest and highest weights.

### MRI

All subjects underwent MRI of the right knee in a 1 T extremity unit (ONI Medical Systems, Wilmington, MA, USA). Coronal T_1_-weighted (T_1_W) spin-echo images (TR 400 ms, TE 15 ms, 4 mm slice thickness, 5 mm gap) were obtained through the knee with a field of view of 16 cm to include the distal femoral and proximal tibial metaphyses. Additionally, we performed a spin-lattice relaxation (T_1_) relaxometry acquisition consisting of seven fast-spin-echo (FSE) acquisitions of varying TR values (TR 350–3000 ms, TE 17 ms, 5 mm slice thickness, 2 mm gap) through the knee. Reproducibility (expressed as percent coefficient of variation) for T_1_ measurements with this scanner using standard phantoms is 2% to 4%.

### Visual assessment

**Fig. 1:**
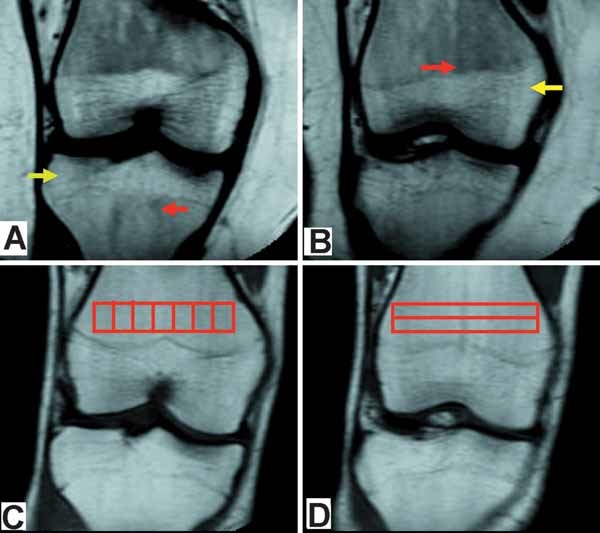
(*A*, *B*) T_1_-weighted MRI images of the right distal femur of a control subject: central slice (*A*) and one slice anterior (*B*). (*C*, *D*) Corresponding images from an anorexic subject. Lower-signal-intensity regions of residual red marrow are much more apparent in the distal femoral metaphyses of the control subject compared with the homogeneous high-signal-intensity fatty marrow seen in the patient with AN. In both groups, T_1_-weighted signal intensity was evaluated in four regions of interest: the medial aspect of the distal femoral metaphysis; the central aspect of the proximal tibial metaphysis, chosen because of striations of hematopoietic marrow evident in controls (*red arrows* in *A* and *B*); and the distal femoral and proximal tibial epiphyses, where yellow marrow was more predominant and the signal intensity more homogeneous (*yellow arrows* in *A* and *B*). The red rectangles in parts (*C*) and (*D*) show the location of the full femoral region of interest. Each rectangle was further subdivided into seven transverse segments extending from lateral to medial (*L* and *M* in *C*) and two horizontal segments, superior and inferior (*D*) for statistical analysis.

Two blinded pediatric radiologists visually assessed all the images for RM content, designated as areas of low signal intensity. Each of the distal femoral and proximal tibial metaphyses was graded according to the following unique scale developed specifically for this study: 0 = homogeneous hyperintensity, no RM; 1 = few hypointense signal areas, mild RM; 2 = scattered hypointense areas, moderate RM; 3 = more diffuse hypointense regions, extensive RM; 4 = all dark, complete RM. Scores were decided by consensus (Fig. 1).

### Relaxometry assessment

T_1_ maps from the T_1_ relaxometry images were generated using a two-parameter-fit iterative algorithm (based on the assumption of monoexponential T_1_ relaxation processes) developed in-house using IDL software (ITT Visual Information Systems, Boulder, CO, USA).

On initial visual inspection of the T_1_W spin-echo images, the control subjects revealed a consistent pattern of lower signal intensity (*red arrows* in Fig. 1*A*, *B*)—presumed to be residual RM—within the medial aspect of the distal femoral metaphysis and the central aspect of the proximal tibial metaphysis, in accordance with previously reported studies.(32) Adolescents with AN showed higher T_1_ signal intensity in these same anatomic locations (see Fig. 1*C*, *D*). The epiphyses, both femoral and tibial, showed uniformly high signal intensity in all subjects, presumed to be YM (*yellow arrows* in Fig. 1*A*, *B*). Based on these observations, four regions of interest were selected—one each in the medial distal femoral metaphysis, the central proximal tibial metaphysis, and the distal femoral and proximal tibial epiphyses. Mean T_1_ values for each region were recorded (Image J, NIH, Bethesda, MD, USA) (see Fig. 1). The anatomic locations of these regions were consistent for all subjects, and all regions of interest were consistently 72 square pixels (approximately 25 mm^2^) in size.

Finally, based on trends seen in the analysis of the four regions of interest described above, T_1_ values were recorded for a larger rectangular region of interest (85 × 17 voxels, approximately 50 × 10 mm in size) for both the AN subjects and controls spanning the full width of the distal femoral metaphysis just above the growth plate, adjacent to the growth plate, and over two consecutive slices (shown in Fig. 1*C*, *middle slice*, and Fig. 1*D*, *next more anterior slice*). To eliminate spurious readings (usually caused by patient motion or magnetic field inhomogeneity), T_1_ values below 200 ms or greater than 1000 ms (2% of the data) were discarded.

### Statistical analysis

Characteristics of the two samples were compared by Fisher exact test for dichotomous and polytomous variables, Wilcoxon rank-sum test for highly skewed measures, and Student's *t* test for other continuous measures. The visual assessments of signal intensity were compared between AN and control by analysis of covariance, adjusting for age. Because these data were mildly skewed, the results were corroborated by Wilcoxon test.

The four-region sample of relaxometric data was analyzed by mixed-model analysis of variance (ANOVA). Mean T_1_ was calculated in each subject's four regions of interest and analyzed separately for femur and tibia. The independent variables were age, AN status, and location (metaphysis or epiphysis), with random effects accounting for correlation within matched pair and subject. A location × AN interaction term was included to test whether the effect of AN status differed between metaphysis and epiphysis.

The full-width femur data were analyzed similarly, with individual voxel T_1_ as dependent variable. Location within the rectangular sampling region was characterized by a seven-segment division extending from medial to lateral (see Fig. 1*C*) and a two-segment division from superior to inferior (see Fig. 1*D*). Independent variables in the ANOVA were age, AN status, MRI slice, sampling segment location, and all interactions among AN, slice, and segment. Random effects were included to account for correlation with matched pair and subject.

In both relaxometric analyses, contrasts of interest and age-adjusted means for each combination of independent variables were constructed from the ANOVA model with standard errors for assessment of statistical significance. A two-tailed *p* value of less than .05 was considered statistically significant. SAS software (Version 9.1, Cary, NC, USA) was used for all computations.

## Results

### Clinical characteristics

Basic demographic and clinical data collected from all subjects are shown in Table 1. Compared with control subjects, the participants with AN had similar age and height but lower weight and BMI. There were no differences between the patients with AN and controls with respect to race, ethnicity, and self-reported fracture history or family history of osteoporosis. There were no significant trends noted in the location of fractures between the two groups. One pathologic fracture was reported: a vertebral compression fracture in a subject with AN. None of the participants had sustained a fracture within 6 months of study participation.

**Table 1:** Characteristics of Anorexic and Control Subjects

	Anorexic (20)	Control (20)	p^a^
	*Mean ± standard deviation*		
Age, years	16.1 ± 1.6	16.3 ± 1.6	.72
Height, cm	164.0 ± 6.5	163.9 ± 5.8	.98
Weight, kg	45.5 ± 4.5	60.0 ± 6.6	<.0001
BMI, kg/m^2^	16.9 ± 1.5	22.3 ± 2.0	<.0001
	*N (%)*		
Race			1
Caucasian	18 (90)	17 (85)	
Asian	2 (10)	2 (10)	
Other	0 (0)	1 (5)	
Ethnicity			1
Hispanic	1 (5)	0 (0)	
Non-Hispanic	19 (95)	20 (100)	
Fracture history			.53
Yes	12 (60)	9 (45)	
No	8 (40)	11 (55)	
Family history of osteoporosis			.13
Yes	8 (40)	2 (10)	
No	10 (50)	15 (75)	
Don't know	2 (10)	3 (15)	
	*Median (minimum–maximum)*		
Past fractures	1 (0–3)	0 (0–4)	.48
Duration of anorexia, months	12 (2–108)	—	
Duration of amenorrhea	8 (3–99)	—	

a From Student's *t* test comparing means, Fisher exact test comparing percentages, or Wilcoxon test comparing medians. *p* = 1 is a valid outcome for Fisher exact test, indicating maximal nonsignificance. Similar results were obtained from paired (age-matched) or age-adjusted analysis.

### Visual assessment of MRI results

Among the 39 subjects with acceptable image quality for visual assessment, adolescents with AN exhibited lower scores than controls (higher T_1_ signal intensity, less RM) in the distal femoral, as well as proximal tibial metaphyses (Table 2). The age-adjusted difference in mean score was nearly twofold at the femur (0.64 versus 1.22, *p* = .03) as well as the tibia (0.54 versus 0.96, *p* = .08).

**Table 2:** Visual Assessment of T_1_ Signal Intensity at Standard Locations in Anorexic and Control Subjects

	Intensity^a^		
		
Location	Anorexic (20)	Control (19)	p^b^
Distal femur metaphysis	0.64 ± 0.18	1.22 ± 0.18	.03
Proximal tibia metaphysis	0.54 ± 0.16	0.96 ± 0.16	.08

a Age-adjusted mean ± standard error, scale from 0 (homogeneous hyperintensity, characteristic of yellow marrow) to 4 (all dark, characteristic of red marrow). One control image was of insufficient quality to assess

b From analysis of covariance comparing anorexic to control, adjusted for age. Corroborated by Wilcoxon test allowing for skewed data.

Visual assessment of the T_1_W spin-echo images also consistently identified residual RM within the medial aspect of the distal femoral metaphysis and the central aspect of the proximal tibial metaphysis in all control subjects, and these areas were further studied using relaxometry data. There was no relation of duration of either AN or amenorrhea to the visual assessment measures (*p* *>* .10).

### MRI relaxometry results

Thirty-six images (18 AN, 18 control) were free of motion or technical problems and were of sufficient quality for the initial relaxometry analysis. Mean T_1_ values were consistently lower in the epiphyses than in the metaphyses for all subjects (controls and patients with AN), by 120 ms in the femur and 70 ms in the tibia, both highly significant (Table 3). Compared with controls, the AN subjects showed higher mean T_1_ in the epiphyses but lower mean T_1_ in the metaphyses. This pattern occurred in both the femur and the tibia (see Fig. 1). The AN-control difference was not statistically significant in any of the four individual regions (see Table 3), but the net AN-control difference between epiphysis and metaphysis was 70 ms and significant in the femur (−35 versus +31 ms, *p* = .02) and of smaller, nonsignificant magnitude but similar pattern in the tibia (−17 versus +11 ms, *p* = .28). The AN-control difference in the distal femur, though not statistically significant (*p* = .12), was largest in estimated magnitude of the four regions studied and led us to examine the T_1_ relaxometry data from the distal femur in a more systematic fashion.

**Table 3:** Relaxometric Assessment of T_1_ Values at Standardized Locations in Femur and Tibia of Anorexic and Control Subjects

	T_1_, ms^a^				
			
Location	All (36)	AN (18)	Control (18)	AN-control, ms^b^	p, AN vs contro
Distal femur					
Epiphysis, lateral	444 ± 57	460 ± 63	429 ± 48	+30.9 ± 21.9	.17
Metaphysis, medial	564 ± 107	547 ± 99	582 ± 114	−35.1 ± 21.9	.12
p, metaphysis versus epiphysis	<.0001			.02	
Proximal tibia					
Epiphysis, lateral	436 ± 54	441 ± 39	430 ± 67	+11.2 ± 21.2	.60
Metaphysis, central	507 ± 82	498 ± 87	515 ± 79	−17.2 ± 21.2	.42
p, metaphysis versus epiphysis	<.0001			.28	

a Mean ± standard deviation. Low values of T_1_ are characteristic of yellow marrow, high values characteristic of red marrow. Two anorexic images and two control images were of insufficient quality to assess

b Difference between anorexic and control means ± standard error estimated by mixed-model analysis of covariance adjusted for age, location, and intrasubject correlation.

**Fig. 2:**
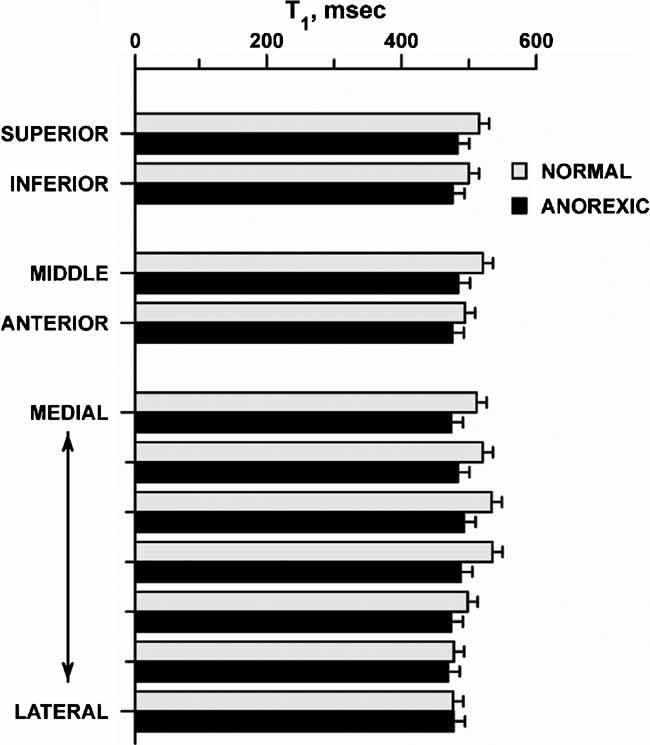
Subjects with AN showed generally lower mean T_1_ values (less red marrow) than controls. Within each slice, the effect was most pronounced in the medial and superior regions. The effect also was greater in the middle slice than in the more anterior slice. Bars indicate mean + 1 SEM.

Relaxometry data from the full width of the femoral metaphyses were more revealing. Thirty-two images (18 AN, 14 control) were of sufficient quality for the full-width analysis. Subjects with AN showed mean T_1_ consistently lower than in controls by 30 to 50 ms in every part of the sampling region with the exception of the lattermost aspect of the metaphysis (Fig. 2). Within the rectangular region of interest, the effect was most pronounced in the medial and superior areas regardless of slice. The effect also was greater in the middle slice compared with the more anterior slice. There was no relation of either duration of AN or amenorrhea to any of the relaxometry measures (*p* *>* .10).

## Discussion

We found significant differences between bone marrow composition within the knee in adolescent girls with AN compared with their healthy counterparts. Both qualitative and quantitative findings support our hypothesis of premature conversion of hematopoietic to fatty marrow in adolescent girls with AN. Blinded visual assessments revealed increased T_1_ signal intensity, presumably related to fat content, most pronounced in the distal femoral metaphyses in girls with AN compared with their healthy counterparts. Similarly, T_1_ values obtained at fixed anatomic locations demonstrated a consistent increase in YM content in the metaphyses of affected patients. These results suggest that young women with this disease experience a striking premature conversion of RM to YM during a developmental period when they should exhibit a more heterogeneous marrow cellularity. Noteworthy is the fact that the bone marrow findings in the participants with AN persisted after accounting for both duration of amenorrhea and disease.

The finding of increased YM among the adolescents with AN is concerning, with potential implications for fracture risk in these patients. There are several explanations as to why increased YM within bone marrow may increase fracture risk. As proposed by Gimble(10) and Okazaki,(33) the process of adipogenesis may compete with osteogenesis, resulting in the differentiation of mesenchymal stem cells into adipocytes over osteoblasts. This may be due to altered hormonal levels, including low leptin and IGF-1 and elevated cortisol.(11) However, other investigators suggest alternate pathways by which increased marrow fat may predispose to bone fragility. Bone fat can contribute to diminished structural bone integrity, resulting in diminished bone mass and therefore reduced skeletal strength.(34) Additionally, the quality of bone marrow itself may be a determinant of mechanical vertebral strength. Therefore, it has been suggested that the presence of fat may reduce the biomechanical strength of bone. Marrow-filled intertrabecular spaces function as “energy dampers” and serve as biomechanical support structures for medullary bone. While RM contributes to hydrostatic strengthening, YM, which is higher in fat content and significantly weaker, causes greater vertebral compressibility, thereby conferring an increased fracture risk.(32)

As exhibited by elderly women who experience increased marrow adipogenesis following menopause, estrogen appears to be an adipogenesis inhibitor.(10)(33) Estrogen and leptin have been suggested as the links between fat and bone, both of which are low in AN, potentially moderating an inverse relationship between mesenchymal stem cell (MSC) differentiation into osteoblasts and adipocytes. High-affinity leptin receptors are present in human mesenchymal stem cells derived from healthy control and osteoporotic donors.(8) Therefore, hormonal changes that ensue in a young women with AN may cause such individuals to experience a “switch” from osteogenesis to adipogenesis, resulting in more lipid accumulation in the bone marrow compared with a healthy adolescent. Mesenchymal stem cells that express the ligand-activated transcription factor peroxisome proliferator–activated receptor λ_2_ (PPARλ_2_), when activated, are likely to commit into the adipocyte lineage.(24) Recent investigations of marrow adiposity and bone mass in healthy adolescent girls demonstrate an inverse relationship between marrow adiposity and bone density in both the axial and appendicular skeleton. This observation supports the hypothesis that a common progenitor cell exclusively differentiates into either the cell lineage responsible for bone formation or that responsible for fat formation.(7)

The finding of increased YM in the bone marrow of adolescent girls with AN is supported by similar observations in animal models. Congenic mice with low IGF-1 levels demonstrated similar physiologic characteristics to humans with AN, with reduced cortical and trabecular bone mineral density and impaired bone formation.(35) Ackert-Bicknell and colleagues showed that these mice have greater *PPARγ* gene expression, a positive regulator of adipogenesis, and a suppressor of osteogenesis.(36) In a separate study investigating the relationship between BMD and bone marrow composition in humans, Schellinger and colleagues(34) reported that although increased fat fraction in bone marrow correlates with structurally weakened bones, it does not necessarily equate with lower BMD. The investigators demonstrated that a majority (73%) of sample subjects with more fragile bones had a bone marrow fat fraction above 54%, although this finding was not significantly correlated with a low BMD. In another model, administration of growth hormone to hypophysectomized rats inhibited the differentiation of stromal cells into adipocytes.(37) These animal data suggest that the characteristic fall in IGF-1 is an important signal for adipogenesis in marrow and in AN may stem from growth hormone resistance, which has been shown to be present in these young women.(38) This hormonal alteration also may play a pathophysiologic role in the marrow changes that are seen in adolescents and young women with AN.

Conflicting conclusions regarding bone marrow adiposity in young women with AN may be due to the fact that different studies have used differing methodologies to evaluate bone marrow, including dual-energy CT,(16) bone marrow biopsy,(31) and magnetic resonance relaxometry, with or without spectroscopy.(15)(17)(18) In addition, conclusions often have been based on the qualitative judgment of radiologists, as well as retrospective chart reviews. In one study, results from dual-energy CT demonstrated disproportionately increased levels of intravertebral fat in patients with AN for their severity of osteopenia, whereas individuals with Cushing syndrome demonstrated no such fat increase.(16) This early study provided proof of concept that patients with AN exhibit increased marrow fat, the etiology of which was not understood. Similarly, Abella and colleagues performed bone marrow biopsies in adolescents and young women with AN.(12) While 20% of patients with AN exhibited complete gelatinous degeneration of bone marrow, 39% of patients with AN had hypoplastic or aplastic bone marrow, consisting of 10% increased marrow fat fraction, compared with that of healthy individuals. The present study is the first to use images obtained via noninvasive MRI in order to both qualitatively and quantitatively assess bone marrow adiposity in adolescents with AN compared with healthy girls. Novel information was obtained on conversion of RM to YM, which represents a normal developmental process and one that appears to be accelerated in AN.

Limitations of this study must be acknowledged and considered. Our sample size was relatively small for this preliminary study. To acquire more conclusive results regarding quantitative MR assessment of marrow fat, future studies of a larger sample size are needed to support these initial results. This study demonstrates the feasibility of using a 1.0 T extremity scanner for relaxometry studies in the musculoskeletal system and, in particular, in girls with AN. Because patients with psychiatric disorders such as AN often exhibit significant anxiety and claustrophobia when scanned in conventional, enclosed MRI scanners, documentation of the capability and reliability of an extremity scanner is of importance. Avoiding an enclosed scanner also greatly increased subject recruitment and the feasibility of this study. Admittedly, a 1.0 T open-magnet configuration has lower resolution and lower signal-to-noise ratio than a higher-field-strength whole-body scanner. However, sufficient resolution and signal-to-noise ratio were achieved via this approach. Since changes in marrow fat appear to be site-specific, correlating measurements in the peripheral versus axial skeleton also will be informative. In contrast to adult studies that have focused primarily on the axial skeleton, our methodology afforded an assessment of the peripheral skeleton, which is highly informative in a pediatric population that is most likely to experience extremity fractures. Data on fracture history, an endpoint of potential clinical importance, was obtained by self-report with inherent limitations, especially in an adolescent patient group. We did not obtain information on menarchal age in the patients or healthy controls. This point should be examined in future studies because some of the differences observed may have been affected by differences in skeletal maturity. With a 1 T scanner, we also were limited in our ability to evaluate cortical thickness and bone size. An important question to address in future studies is whether parameters of bone size are related to marrow composition. Lastly, we did not have bone density or laboratory data to correlate with our imaging findings. Therefore, explanations for the marrow findings are speculative at this point in the absence of hormonal measurements, which will be important to obtain in future protocols.

In summary, it has been documented that girls suffering from AN experience early bone loss, resulting in heightened risk for bone fracture and early osteoporosis. We observed a higher fatty marrow content within the metaphyses of the distal femur and proximal tibia in adolescent girls with AN compared with healthy age- and race-matched control subjects. These MRI findings suggest premature conversation of RM to YM in adolescents suffering from this eating disorder. Given the known hormonal abnormalities in affected individuals, these changes may accelerate adipocyte conversion over osteoblast differentiation in the mesenchymal stem cell pool, resulting in increased bone marrow adipogenesis and premature conversion to YM. These marrow findings are important clinically because they may mediate, in part, the early osteoporosis and heightened fracture risk seen in these young patients.

## References

[1] Gordon CM, Goodman E, Emans SJ (2002). Physiologic regulators of bone turnover in young women with anorexia nervosa. J Pediatr..

[2] Soyka LA, Grinspoon S, Levitsky L, Herzog DB, Klibanski A (1999). The effects of anorexia nervosa on bone metabolism in female adolescents. J Clin Endocrinol Metab..

[3] Bachrach LK, Guido D, Katzman D, Litt IF, Marcus R (1990). Decreased bone density in adolescent girls with anorexia nervosa. Pediatrics.

[4] Rigotti NA, Neer RM, Skates SJ, Herzog DB, Nussbaum SR (1991). The clinical course of osteoporosis in anorexia nervosa. JAMA..

[5] Golden NH (2007). Eating disorders in adolescence: what is the role of hormone replacement therapy?. Curr Opin Obstet Gynecol..

[6] Misra M (2008). Long-term skeletal effects of eating disorders with onset during adolescence. Ann NY Acad Sci..

[7] Di Iorgi N, Rosol M, Mittelman SD, Gilsanz V (2008). Reciprocal relation between marrow adiposity and the amount of bone in the axial and appendicular skeleton of young adults. J Clin Endocrinol Metab..

[8] Hess R, Pino A, Rios S, Fernandez M, Rodriguez J (2004). High- affinity leptin receptors are present in human mesenchymal stem cells (MSCs) derived from control and osteoporotic donors. J Cell Biochem..

[9] Gimble JM, Nuttall ME (2004). Bone and fat: old questions, new insights. Endocrine..

[10] Gimble JM, Zvonic S, Floyd E, Kassem M, Nuttall M (2006). Playing with bone and fat. J Cell Biochem..

[11] Shapses SA, Riedt CS (2006). Bone, body weight and weight reduction: what are the concerns?. J Nutr..

[12] Abella E, Feliu E, Granada I (2002). Bone marrow changes in anorexia nervosa are correlated with the amount of weight loss and not with other clinical findings. Am J Clin Pathol..

[13] Vande Berg BC, Malghem J, Devuyst O, Maldague B, Lambert MJ (1994). Anorexia nervosa: correlation between MR appearance of bone marrow and severity of disease. Radiology.

[14] Vande Berg BC, Malghem J, Lecouvet FE, Lambert M, Maldague BE (1996). Distribution of serous like bone marrow changes in the lower limbs of patients with anorexia nervosa: predominant involvement of the distal extremities. AJR..

[15] Geiser F, Mürtz P, Lutterbey G (2001). Magnetic resonance spectroscopic and relaxometric determination of bone marrow changes in anorexia nervosa. Psychosom Med..

[16] Mayo-Smith W, Rosenthal DI, Goodsitt MM, Klibanki A (1989). Intravertebral fat measurement with quantitative CT in patients with Cushing disease and anorexia nervosa. Radiology.

[17] Okamoto K, Ito J, Ishikawa K, Sakai K, Tokiguchi S (2001). Change in signal intensity on MRI of fat in the head of markedly emaciated patients. Neuroradiology.

[18] Demaerel P, Daele MCV, De Vuysere S, Wilms G, Baert AL (1996). Orbital fat edema in anorexia nervosa: a reversible finding. AJNR..

[19] Bredella MA, Fazeli PK, Miller KK (2009). Increased bone marrow fat in anorexia nervosa. J Clin Endocrinol Metab..

[20] Custer RP, Ahfeldt FE (1932). Studies on the structure and function of bone marrow: II. Variations in cellularity in various bones with advancing years of life and their relative response to stimuli. J Lab Clin Med..

[21] Vost A (1963). Osteoporosis: a necropsy study of vertebrae and iliac crests. Am J Pathol..

[22] Hartsock RJ, Smith EB, Petty CS (1965). Normal variations with aging of the amount of hematopoietic tissue in bone marrow from the anterior iliac crest: a study made from 177 cases of sudden death examined by necropsy. Am J Clin Pathol..

[23] Meunier P, Aaron J, Edouard C, Vignon G (1971). Osteoporosis and the replacement of cell populations of the marrow by adipose tissue: a quantitative study of 84 iliac bone biopsies. Clin Orthop Relat Res..

[24] Duque G (2003). Will reducing adipogenesis in bone increase bone mass? PPARγ_2_ as a key target in the treatment of age-related bone loss. Drug News Perspect..

[25] Lang P, Steiger P, Faulkner K, Gluer C, Genanth K (1991). Osteoporosis: current techniques and recent development in quantitative bone densitometry. Radiol Clin North Am..

[26] Dooms GC, Fisher MR (1985). Bone marrow imaging: magnetic resonance studies related to age and sex. Radiology.

[27] Demmler K, Burkhardt R (1978). Relations between fatty tissue, cancellous bone and vascular pattern of the iliac bone in aplastic anaemia. Bibl Haematol..

[28] Justesen J, Stenderup K, Ebbesen EN, Mosekilde L, Steiniche T, Kassem M (2001). Adipocyte tissue volume in bone marrow is increased with aging and in patients with osteoporosis. Biogerontology.

[29] Kaarian L, Graves G (1977). Compressive strength characteristics of the human vertebral column. Spine..

[30] Nuttall ME, Gimbel JM (2000). Is there a therapeutic opportunity to either prevent or treat osteopenic disorders by inhibiting marrow adipogenesis?. Bone..

[31] Devuyst O, Lambert M, Rodhain J, Lefebvre C, Coche E (1993). Haematological changes and infectious complications in anorexia nervosa: a case-control study. Q J Med..

[32] Moore SG, Dawson KL (1990). Red and yellow marrow in the femur: age-related changes in appearance at MR Imaging. Radiology.

[33] Okazaki R, Inoue D, Shibata M (2002). Estrogen promotes osteoblast differentiation and inhibits adipocyte differentiation in mouse bone marrow stromal cell lines that express estrogen receptor (ER) alpha or beta. Endocrinology.

[34] Schellinger D, Lin CS, Lim J, Hatipoglu HG, Pezzullo JC, Singer AJ (2004). Bone marrow fat and bone mineral density on proton MR spectroscopy and dual-energy X-ray absorptiometry: their ratio as a new indicator of bone weakening. AJR..

[35] Rosen CJ, Ackert-Bicknell CL, Adamo ML (2004). Congenic mice with low serum IGF-I have increased body fat, reduced bone mineral density, and an altered osteoblast differentiation program. Bone..

[36] Ackert-Bicknell CL, Demissie S, Marín de Evsikova C (2008). PPARγ by dietary fat interaction influences bone mass in mice and humans. J Bone Miner Res..

[37] Appiagyei-Dankah Y, Tapiador CD, Evans JF, Castro-Magana M, Aloia JF, Yeh JK (2003). Influence of growth hormone on bone marrow adipogenesis in hypophysectomized rats. Am J Physiol Endocrinol Metab..

[38] Misra M, Miller KK, Bjornson J (2003). Alterations in growth hormone secretory dynamics in adolescent girls with anorexia nervosa. J Clin Endocrinol Metab..

[39] Michaelsson K, Bergstrom R, Mallmin H, Holmberg L, Wolk A, Ljunghall S (1996). Screening for osteopenia and osteoporosis: selection by body composition. Osteoporos Int..

